# Current Perspective on Retinal Migraine

**DOI:** 10.3390/vision5030038

**Published:** 2021-08-23

**Authors:** Yu Jeat Chong, Susan P. Mollan, Abison Logeswaran, Alexandra B. Sinclair, Benjamin R. Wakerley

**Affiliations:** 1 Birmingham Neuro-Ophthalmology, Ophthalmology Department, Queen Elizabeth Hospital, University Hospitals Birmingham NHS Foundation Trust, Birmingham B15 2TH, UK; yu.chong.2@city.ac.uk (Y.J.C.); soozmollan@doctors.org.uk (S.P.M.); 2 Moorfields Eye Hospital NHS Foundation Trust, London EC1V 2PD, UK; abison.logeswaran.1@city.ac.uk; 3 Department of Neurology, University Hospitals Birmingham NHS Foundation Trust, Birmingham B15 2TH, UK; a.b.sinclair@bham.ac.uk; 4 Metabolic Neurology Group, University of Birmingham, Edgbaston, Birmingham B15 2TT, UK

**Keywords:** retinal migraine, ocular migraine, ophthalmic migraine, migraine aura

## Abstract

Retinal migraine was first formally described in 1882. Various terms such as “ocular migraine” and “ophthalmic migraine” have since been used interchangeably in the literature. The lack of a consistent consensus-based definition has led to controversy and potential confusion for clinicians and patients. Retinal migraine as defined by the International Classification of Headache Disorders (ICHD) has been found to be rare. The latest ICHD defined retinal migraine as ‘repeated attacks of monocular visual disturbance, including scintillation, scotoma or blindness, associated with migraine headache’, which are fully reversible. Retinal migraine should be considered a diagnosis of exclusion, which requires other causes of transient monocular visual loss to be excluded. The aim of this narrative review is to summarize the literature on retinal migraine, including: epidemiology and risk factors; proposed aetiology; clinical presentation; and management strategies. It is potentially a misnomer as its proposed aetiology is different from our current understanding of the mechanism of migraine

## 1. Introduction and Historical Evolution

There has been extensive documentation of migraine as a disorder, dating back to over 4000 years ago from ancient Mesopotamia [1]. One of the earliest descriptions of visual symptoms associated with migraine was a case report by Hippocrates (c. 460—c. 370 BC) in ancient Greece [1]:

‘he seemed to see something shining before him like a light, usually in part of the right eye; at the end of a moment, a violent pain supervened in the right temple, then in all the head and neck. vomiting, when it became possible, was able to divert the pain and render it more moderate.’

In 1882, Galezowski first used the term *ophthalmic megrim* to describe a case series of four patients with permanent monocular visual loss and migraine headaches. He hypothesized that *ophthalmic megrim* is a condition that may ‘*occasionally lead to organic changes in the retina or retinal vessels*’, with findings such as retinal thrombosis and optic disc atrophy [2].

The term retinal migraine was first introduced by Caroll in 1970 when he suggested that although controversial, it could *‘possibly be applied to uncommon cases’* of migraine [3]. He presented a case series of 15 patients with monocular visual loss without associated features of headache, mostly transient with a duration of less than 10 min and rarely lasting more than an hour. A few cases had permanent visual loss resulting in optic atrophy, which he hypothesized was secondary to raised intraocular pressure from paroxysmal vasoconstriction of the central retinal artery or the ophthalmic artery.

Without an internationally agreed definition, authors have used various terms interchangeably to describe monocular visual loss, which is either: associated with headache, not associated with headache, transient, or permanent. These terms include ‘ophthalmic migraine’, ‘ocular migraine’, and anterior pathway migraine [4,5,6,7].

Retinal migraine was first included in the 1988 edition of the International Classification of Headache Disorders (ICHD) with an internationally agreed definition [8]. In the latest 2018 ICHD-3, retinal migraine is defined as ‘repeated attacks of monocular visual disturbance, including scintillation, scotoma, or blindness, associated with migraine headache’ [9]. These episodes are fully reversible. The full diagnostic criteria of retinal migraine are included in Box 1.

In this narrative review, we have summarized the literature on retinal migraine as defined by the latest ICHD, to include epidemiology and risk factors, proposed aetiology, clinical presentation, and management strategies.

Box 1 **ICHD classification of retinal migraine [9].**

1. Attacks fulfilling criteria for migraine with aura and the criterion below

2. Attacks fulfilling criteria for migraine without aura and the criterion below

3. Aura characterized by both of the following:

(A) Fully reversible, monocular, positive, and/or negative visual phenomena (e.g., scintillations, scotomata or blindness) confirmed during an attack by either or both of the following:

- clinical visual field examination
- the patient’s drawing of a monocular field defect (made after clear instruction)

(B) At least two of the following:

- spreading gradually over ≥5 min
- symptoms last 5–60 min
- accompanied, or followed within 60 min, by headache

4. Other causes of amaurosis fugax were excluded, and an episode cannot be accounted for by another ICHD-3 diagnosis. This is a diagnosis of exclusion.

The ICHD-3 criteria has to meet points 1, 3(A), 3(B), and 4; while the ICHD-2 criteria has to meet points 2, 3(A), and 4 for a diagnosis of retinal migraine to be made. In the ICHD-2, it was specified that there should be normal ophthalmological examinations between attacks.

## 2. Methods

A narrative review was conducted based on the Scale for the Assessment of Narrative Review Articles (SANRA) principles due to its broad scope and ability to include a range of information sources. The following databases were searched by the authors Y.Y.C. and A.L.: Embase Classic, Embase, and Ovid MEDLINE. We used the search term ‘retinal migraine’ and identified 270 articles.

All studies included had a clear research question, appropriate study design, and discussion of results. Non-English articles and conference proceedings were excluded. Additional articles that were relevant were hand-searched from Google Scholar and also identified from citations within articles.

## 3. Epidemiology and Risk Factors

Retinal migraine is a rare condition and its true incidence is difficult to ascertain due to a number of factors. Firstly, a consensus-based diagnostic criteria was only established in the 1988 ICHD. The 1988 ICHD defined retinal migraine as ‘*at least two attacks’* of fully reversible monocular visual disturbance with typical migraine headache, normal neuro-ophthalmic examination, and exclusion of other causes [8].

Secondly, the diagnostic criteria of retinal migraine has continued to evolve with time. In 2004, the revised ICHD-2 maintained the same diagnostic criteria, with the difference that it is associated with headaches that fulfill the criteria for migraine without aura [10]. Grosberg et al., in 2006, summarized the clinical features and prognosis of the largest case series of retinal migraine patients (40 cases from previous literature, with 6 new cases) based on the ICHD-2 criteria [11]. Contrary to the ICHD-2 criteria of migraine without aura, they showed that half of the patients meeting the definition of retinal migraine had a history of migraine with aura. The 2018 ICHD-3 criteria was subsequently updated to ‘*attacks fulfilling criteria for migraine with aura’* [9].

The same authors also showed that out of the 46 patients with retinal migraine, 28 (61%) were female, with the age of onset ranging from 7 to 54 years old [11]. They further subcategorized patients based on whether they only showed transient visual loss (TVL), or subsequently developed permanent visual loss (PVL). Twenty one out of 46 (46%) patients experienced PVL, with a preponderance for females (15 females compared to 6 males). The mean age of onset was similar overall in both groups, at 24.7 years for the TVL group and 23.0 years for the PVL group. In females, the peak age of onset was the third and fourth decade for the TVL group, and second and third decade for the PVL group. A family history of migraine was documented in 14 out of 46 (30%) of patients.

Thirdly, there are discrepancies in the literature with regards to inclusion criteria for retinal migraine. Another literature review based on the ICHD-2 criteria was conducted by Hill et al. in 2007, which included patients from Grosberg et al. in 2006 [12]. Of the 142 patients identified, 103 had transient monocular vision loss (TMVL) attributed to retinal migraine. Of the 103, only 16 had clinical manifestations for definite retinal migraine with at least two episodes of TMVL and headaches with a migraine-like phenotype. Of these 16, only five met the full ICHD-2 criteria for retinal migraine. Six out of 16 were female, with a mean age of 25.5 years (ranging from 9 to 53 years).

Discrepancies between Grosberg et al. in 2006 and Hill et al. in 2007 are based on several points of contention. Grosberg et al. included patients who had a single episode of fully reversible monocular visual phenomena occurring during or within 60 min of a migraine attack; while Hill et al. only included patients with two episodes of transient monocular visual loss and headaches suggestive of migraine. Hill et al. also disagreed with the basis of implicating migraine as the cause of PVL, arguing that most patients included by Grosberg et al. with retinal migraine did not have IHS-defined retinal migraine before visual loss. They suggested that the heterogeneity of lesions reported presume retinal migraines such as arterial and vein occlusions, localized retinal ischaemia, and optic atrophy cannot be explained by a single unifying mechanism.

An up-to-date review in 2021 by Maher et al. looking at the literature of the past 15 years between 2006 and 2020 identified only 12 cases of retinal migraine based on the ICHD-3 criteria [13]. Ten out of 12 were female with an average age of 39.5 years. Eleven out of 12 had a prior history of migraine headaches, with four reporting previous history of migraine with aura; although the characteristic of migraine with or without aura was only noted in seven individuals.

The paucity of existing literature and lack of consensus regarding the definition and inclusion criteria of retinal migraine patients makes our understanding of epidemiology and risk factors of this condition challenging.

## 4. Proposed Aetiology

During early embryonic development, the retina and optic nerve emerge from the diencephalon and are considered as part of the central nervous system [14]. The retina is composed of different layers of specialized neurons, including retinal ganglion cells (RGCs), the axons of which form the optic nerve. The axons of the optic nerve continue to synapse at the lateral geniculate nucleus in the thalamus and then finally to the superior colliculus in the midbrain as part of the higher cortical visual processing centres, which allow humans to perceive images of the world [15].

When Carroll described the term retinal migraine in the 1970s, he hypothesized that the mechanism of visual loss is due to paroxysmal constriction or spasm in either the central retinal artery or ophthalmic artery [3]. Hill et al., in 2007, argued that most cases of monocular vision loss purported to be retinal migraine did not meet the ICHD criteria, and could instead be diagnosed as ‘presumed retinal vasospasm’. In his editorial, Winterkorn (2007) further added that ‘*retinal migraine is an oxymoron’* because the notion that a migraine can occur in the retina was derived from the outdated vasospastic theory of migraine [16]. The vascular or vasospastic hypothesis of migraine has been largely refuted by advances in intracerebral blood flow imaging [17]. While we still do not fully understand migraine, advances in the understanding of its pathophysiology indicate a complex interplay of mechanisms involving hypothalamic activation, alteration in thalamo-cortical circuits, brainstem activation, cortical spreading depolarisation, release of chemicals such as calcitonin gene-related peptide (CGRP), and pituitary adenylate cyclase activating polypeptide (PACP) [18]. A study that mapped out the visual precept of aura in a migraine showed that these attacks tend to originate in the central visual field, before spreading peripherally, with a speed of 2–3 mm/min. This further suggests the involvement of the visual cortex [19]. If we accept that the visual aura is a cortical process that results in binocular symptoms, the term retinal migraine becomes potentially confusing and anatomically incongruous. We are referring to cortical mechanisms as though they are occurring in the retina [16].

Kosmorsky argued that there is no definitive way to tell if a vasospastic process is secondary to focally-generated chemicals or mediated neuronally from migraine [20]. He demonstrated a case of a 30-year-old women with 8–10 episodes of recurrent symptoms of flashing lights, grey out, and loss of vision leading to no perception of light (NPL), which was solely monocular to the right eye and in the absence of headache. Fundus fluorescein angiography (FFA) performed while she was symptomatic with NPL vision (corroborated with an afferent pupillary defect) showed delayed central retinal artery circulation with normal choroidal circulation. Within 2 h, she completely regained her vision in the right eye. A repeat FFA 2 days later was normal and other investigations did not identify a cause for her monocular visual loss. Although this case did not meet the ICHD criteria of retinal migraine, the author explained that focal vasospasm alone as an explanation was inadequate as one would not expect circulating vasoactive molecules to induce spasm in the same vascular distribution.

To further support the theory that retinal migraine is a distinct entity from retinal vasospasm, Ota et al. recorded a fundus video of a 29-year-old women with a 10-year history of migraine with aura, and monocular symptoms meeting the ICHD-3 criteria, while the patient was symptomatic [21]. The 1 min and 55 s video showed dynamic changes in the fundus over time, with narrowing of retinal arteries and veins initially, and blood columns in the veins interrupted with a rouleaux formation, pale optic disc, and dark choroid. The narrowing of the retinal vessels and disc pallor diminished gradually during the course of the video, with dilated retinal vessels and hyperaemic disc after 1 min and 28 s. They speculated that the ophthalmic artery was the main component of vasoconstriction, with vasodilation at the end of the attack representing compensation for retinal hypoxia.

Lastly, several authors have proposed the cortical spreading depression (CSD) theory of retinal neurons as a plausible explanation [5,13,22]. Cortical spreading depression was first described by Leao in the cortex of rabbits, whereby a slow propagating wave of neuronal depolarization through grey matter is followed by depression of neuronal activity [23]. CSD is one of the purported biological mechanisms of the visual symptoms experience in patients with migraine with aura, although this has not directly been demonstrated in humans to date. An initial brief hyperaemic phase is followed by prolonged oligaemia, starting in the occipital lobe and spreading rostrally across the cortex [23]. This is supported by evidence that SD occurs in chicken retina involving CGRP receptors [24,25].

## 5. Clinical Presentation

Visual disturbances in the context of headache and ocular pain are common [26]. The important distinction is in the characterisation of the visual disturbance and associated visual symptoms [27]. The most important distinguishing feature for a diagnosis of retinal migraine compared to visual aura experienced in those with migraines lies in its strict monocular presentation. In migraine with aura, visual symptoms often present as complex patterns, such as: fortification spectra (zig zag figure near the point of fixation with an angulated scintillating edge), followed by variable degrees of relative scotoma, typically lasting between 5 to 60 min [9,27]. The binocular and homonymous nature of these symptoms often lead to incorrect descriptions by patients, attributing homonymous visual phenomena as monocular. This relates to the greater relative size of the temporal visual fields (extending out to 90 degrees) compared to the nasal visual field (extending out to 60 degrees), resulting in attribution of monocular field loss in the eye with the temporal defect [28].

In contrast with migraine with visual aura, most retinal migraine cases reported to date present with negative symptoms (such as visual field defects including central scotoma, altitudinal visual loss, or sectorial loss): shown by 12 out of 16 cases by Hill et al., and 11 out of 12 cases by Maher et al.

Retinal migraine is a diagnosis of exclusion only after other causes of TMVL have been excluded. In particular, conditions such as amaurosis fugax, carotid artery occlusive disease, and arteritic disease such as giant cell arteritis (GCA) should be considered.

In amaurosis fugax, interrupted blood flow to the central retinal artery or ophthalmic artery can cause symptoms of a blackout in the whole or half of the visual field. Patients can report a ‘curtain’ sweeping in from one side of the vision. This is temporary and typically resolves within minutes and lasts no longer than an hour. It can be caused by thromboembolism originating from an atherosclerotic plaque in the internal carotid artery, or an embolus originating from the heart or aorta. When associated with atheromatous disease, there is a 2% risk of recurrent stroke at 1 year; while those with severe internal carotid artery stenosis have an increased risk of ipsilateral stroke of up to 16% after 3 years [29]. An embolus can also originate from a carotid artery dissection. Arteritis such as GCA is another serious cause of TMVL that can be associated with headache [30].

Other causes of TMVL include ocular diseases such as dry eyes and intermittent angle closure glaucoma; optic nerve disorders such as papilloedema with transient visual obscurations; and other vascular diseases such as choroidal ischaemia from the posterior ciliary arteries. As there are a number different diagnoses for TMVL, patients presenting these symptoms should be examined by an ophthalmologist.

## 6. Investigation

In the context of a patient presenting with symptoms fulfilling the ICHD-3 criteria for retinal migraine, the first priority is to perform the necessary investigations to rule out other causes of TMVL as shown in Table 1. This includes a detailed medical history from the patient, specifically the characteristic, frequency, and duration of vision loss. It would also be helpful to discern if the patient has a history of migraine with or without aura. Following this, patients should undergo a thorough ophthalmological examination including formal visual fields, ideally with documentation of the fundus with photography and optical coherence tomography (OCT). It may be prudent to perform baseline OCT angiography at the disc and within the macular region, should the opportunity arise in the future to capture an episode. To exclude other vaso-occlusive disease, carotid Doppler imaging may be used, and where arteritic processes are involved, inflammatory markers should be evaluated and specific features of the history be excluded [31].

## 7. Management Strategies

Given the few numbers of cases in the existing literature, evidence-based treatment options for retinal migraine are limited. There have been different case reports advocating the use of migraine-based therapies such as propranolol, imipramine, nortriptyline, and verapamil [11,32,33]. Some authors have suggested not using beta-blockers due to the theoretical risk of arteriolar constriction [33]. In others, the symptoms of retinal migraine are managed conservatively by observations alone [21,34].

## 8. Conclusions

Retinal migraine is a very rare cause of TMVL. Newer imaging modalities, such as OCT and OCT angiography, may in the future help provide better understanding and quantification of the retinal and vascular changes that have previously been reported. As retinal migraine is a diagnosis of exclusion, patients who present with TMVL need extensive work to exclude other causes of monocular vision loss. Future research in this distinct clinical entity is needed, as the term “retinal migraine” may be a misnomer that confuses clinicians and patients alike.

## Figures and Tables

**Table 1:** Examples of differential diagnosis of TMVL.

Disease Category	Examples
Vascular disease	Carotid artery disease (amaurosis fugax)
	Giant Cell Arteritis
	Central retinal artery occlusion
	Choroidal ischaemia from posterior ciliary arteries
	Other arteritis processes that affect the short posterior ciliary arteritis
Ocular disease	Dry eyes
	Intermittent angle closure glaucoma
Optic nerve disorders	Papilloedema with transient visual obscuration
	Uhthoff’s phenomenon from demyelinating disease
	Optic nerve compression (either gaze evoked visual loss or transient visual obscuration)

## Data Availability

Not applicable.
